# Self-Healing Properties of Crosslinked PMMA-DVB Copolymer Microcapsules Based on Interfacial Polymerization

**DOI:** 10.3390/polym17050569

**Published:** 2025-02-21

**Authors:** Xiaowei Jiang, Chengwu Tang, Jiachuan Yu, Yuankai Zhou, Xue Zuo

**Affiliations:** School of Mechanical Engineering, Jiangsu University of Science and Technology, Zhenjiang 212003, China; jiangxiaowei@just.edu.cn (X.J.); tangchengwu2025@163.com (C.T.); jiachuanyu@just.edu.cn (J.Y.)

**Keywords:** interfacial polymerization, self-healing, free radical polymerization, microcapsule, coating

## Abstract

To address the issue of metal corrosion caused by microcracks in the coating on the steel structures of offshore drilling platforms, this study employs interfacial polymerization to prepare microcapsules with self-healing functionality for coatings. The microcapsules are fabricated through free radical polymerization between methyl methacrylate (MMA) and ammonium persulfate (APS), along with crosslinking reactions involving divinylbenzene (DVB). The particle size distribution and surface morphology of the microcapsules were optimized by adjusting process parameters using optical microscopy and scanning electron microscopy. Fourier-transform infrared spectroscopy (FT-IR) and thermogravimetric analysis (TGA) were used to characterize the chemical structure and thermal stability of the microcapsules. The results show that when polyvinyl alcohol is used as the emulsifier, the oil–water ratio was 7.5:200, the amount of emulsifier was 1 wt%, the emulsification speed was 2500 r/min, the amount of initiator was 2 g, the core-to-wall ratio was 4:1, and the ambient temperature was 60 °C showed good sphericity, the microcapsules prepared under the optimized parameters exhibit good sphericity, a smooth surface, and an average particle size of 35.17 μm. They have a good core material encapsulation effect and thermal stability, which impart excellent self-healing properties to the epoxy coating. Such microcapsules have promising applications in mitigating the problem of metal corrosion of coatings due to microcracks and improving the service life and reliability of equipment.

## 1. Introduction

Offshore drilling platforms are exposed to salt-containing and high-humidity environments for a long period of time and face serious metal corrosion problems [1,2]. In order to cope with these problems, anti-corrosion coatings are usually applied to the platform’s steel structure to slow down the corrosion process [3]. However, the coating is prone to microcracking during service due to mechanical stress caused by deformation of the mechanical structure [4], aging caused by ultraviolet rays [5], etc., exposing the metal substrate and forming seawater infiltration channels, thereby accelerating metal corrosion. As corrosion intensifies, the coating may peel off, leading to a failure of its protective function [6].

The microcapsule-based self-healing coating offers a novel approach to coating repair [7]. This method endows the coating with self-repairing capabilities by encapsulating repair materials, such as repair agents, within capsules [8,9,10]. When the coating surface cracks or is damaged, the repair material in the microcapsules is released to fill the damaged areas. By promptly repairing the damage to the coating, it can effectively prevent the expansion of corrosion caused by microcracks, scratches, or localized damage [11]. When applied in harsh marine environments, it can enhance the durability and reliability of the coating [12].

Currently, the commonly used methods for preparing self-healing microcapsules include interfacial polymerization [13], in situ polymerization [14], emulsion polymerization. [15]. Among them, the interfacial polymerization method is a technique for preparing microcapsules by separately dissolving monomers in oil and water phases, promoting the emulsification of the oil–water two phases with emulsifiers, and utilizing the polymerization reaction at the oil–water interface to form microcapsule shells [16]. At present, several studies and applications of microcapsules prepared by interfacial polymerization have been explored in the field of coatings. For example, Attaei et al. [17] used isophorone diisocyanate as the core material to prepare and characterize microcapsules, verifying their self-healing effect on coatings. Yuan et al. [18] prepared microcapsules with ethylenediamine as the core material and epoxy resin as the wall material, studied the effects of preparation factors on the morphology of the microcapsules, and tested the environmental resistance of the microcapsules. Mytara et al. [19] prepared fully aliphatic polyamide microcapsules using ethylenediamine and sebacoyl chloride, analyzing the effects of key process parameters on the microcapsules during polymerization. Du et al. [20] prepared double-layer self-healing microcapsules with isocyanate as the core material and polyurea and melamine resin as the shell materials, studied the mechanical properties of the microcapsules, and characterized the self-healing properties of the microcapsule coating using optical microscopy. Additionally, Yuan et al. [21] prepared double-layer microcapsules using phenolic resin and polyurethane as the shell materials and isophorone diisocyanate as the core material, with comprehensive characterization of the physical and chemical properties of the microcapsules. The above research indicates that the core materials currently used for preparing microcapsules are mainly limited to isocyanates and ethylenediamines, while the wall materials are mostly polyurethane and melamine resin types. As healing agents for microcapsules, ethylenediamine, and isocyanate compounds provide excellent self-healing properties; however, their toxicity and volatility may pose potential risks to the environment and to personnel handling these materials [22,23]. As shell materials for microcapsules, polyurethane, and melamine resins can provide good mechanical properties, but they have shortcomings in UV resistance and corrosion resistance [24]. Especially under long-term exposure to ultraviolet radiation and salt mist conditions in the marine environment, degradation may occur, weakening the protective and self-repairing functions of the coating [25].

In this study, a novel self-healing microcapsule was prepared using PMMA-DVB polymer, known for its excellent mechanical properties as the shell material, and EPR resin, which is non-toxic, exhibits good film-forming ability and weather resistance and has excellent compatibility with epoxy resin, as the core material. This microcapsule can effectively enable self-healing functionality in coatings. Through examining the influence patterns of process parameters on microcapsule properties, the optimal parameters for microcapsule preparation were identified. The particle size distribution, surface morphology, chemical structure, thermal stability, and self-healing performance of the microcapsules prepared under these optimized parameters were then tested and characterized. The successful preparation of this microcapsule enriches the types of microcapsules for self-healing coatings, provides a basis for the preparation of microcapsules for self-healing coatings, and is of great significance for enhancing the service life of surface coatings on steel structures of offshore drilling platforms.

## 2. Materials and Methods

### 2.1. Materials

The raw materials and their purity specifications selected for microcapsule preparation are as follows: methyl methacrylate (MMA, analytical grade, Xilong Scientific Co., Ltd., Shantou, China), divinylbenzene (DVB, 80%, Macklin Biochemical Co., Ltd., Shanghai, China), ammonium persulfate (APS, 98%, Xilong Scientific Co., Ltd.), butyl acetate (BA, analytical grade, Xilong Scientific Co., Ltd.), deionized water (Grade I, Weilit Environmental Technology Co., Ltd., Suzhou, China), polyvinyl alcohol (PVA-205, 99%, Kuraray Co., Ltd., Osaka, Japan), and epoxy-modified polyester resin (EPR, molecular weight 1500~3000, SOLTER, Korbach, Germany).

### 2.2. Principle of Microcapsule Preparation

**Figure 1:**
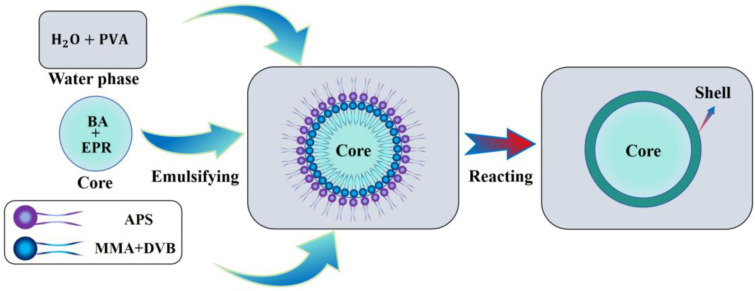
Schematic diagram of microcapsule preparation principle.

Interfacial polymerization is a technique used to prepare microcapsules through a polymerization reaction at the interface between two phases. This method typically involves two immiscible liquid phases: one is an aqueous phase containing water-soluble monomers, and the other is an oil phase containing oil-soluble monomers. When these two phases come into contact, the monomers react at the oil–water interface, forming a polymer shell that is insoluble in either phase, encapsulating the core material inside. The preparation principle of microcapsules based on crosslinking free radical polymerization of MMA and DVB under the action of APS is shown in Figure 1. In this process, MMA acts as the shell material monomer, DVB as the crosslinking agent, and APS as the initiator for the reaction. After the continuous phase emulsion is formed, MMA and DVB monomers are dispersed in small oil droplets. When an appropriate amount of APS dissolves in water, the sulfate radicals generated by the thermal decomposition of APS diffuse to the oil–water interface, initiating the polymerization reaction of MMA and DVB monomers at the interface. As the polymerization progresses, the polymer chains gradually grow and crosslink at the oil–water interface, forming a dense polymer shell that encapsulates the core material in the oil phase, ultimately resulting in microcapsules with a good encapsulation effect.

### 2.3. Microcapsule Preparation Process

First, an appropriate amount of BA, MMA, and DVB is drawn using a rubber bulb pipette and mixed thoroughly. Next, a measured quantity of EPR is added gradually while stirring the mixture with a thermostatic magnetic stirrer for 10 min until fully dissolved, forming the oil phase. Then, a suitable amount of PVA-205 is dissolved in 200 mL of deionized water and stirred for 10 min using a constant temperature magnetic stirrer (TMS, Fangke Instruments Co., Ltd., Changzhou, China, Model: XMTD-702) to ensure complete dissolution, forming the water phase. In addition, an appropriate amount of APS is dissolved in 10 mL of deionized water to prepare a saturated aqueous solution of APS for later use. The oil phase is then added dropwise into the water phase with a rubber bulb pipette and emulsified using a high-shear mixer (HSM, Yineng Experimental Instrument Factory, Changzhou, China, Model: FSH-2A) at a set speed for 2.5 min to produce a stable emulsion. Then the beaker containing the emulsion is transferred to the evaporating dish on the constant temperature magnetic stirrer, the emulsion is heated to 60 °C using a water bath, a saturated aqueous solution of APS is added with a rubber bulb pipette and stirred continuously at a speed of 600 r/min to ensure the initiator is fully dispersed in the emulsion system. After a continuous reaction for 2.0 h, the reaction is stopped by cooling, resulting in a suspension containing microcapsules.

**Figure 2:**
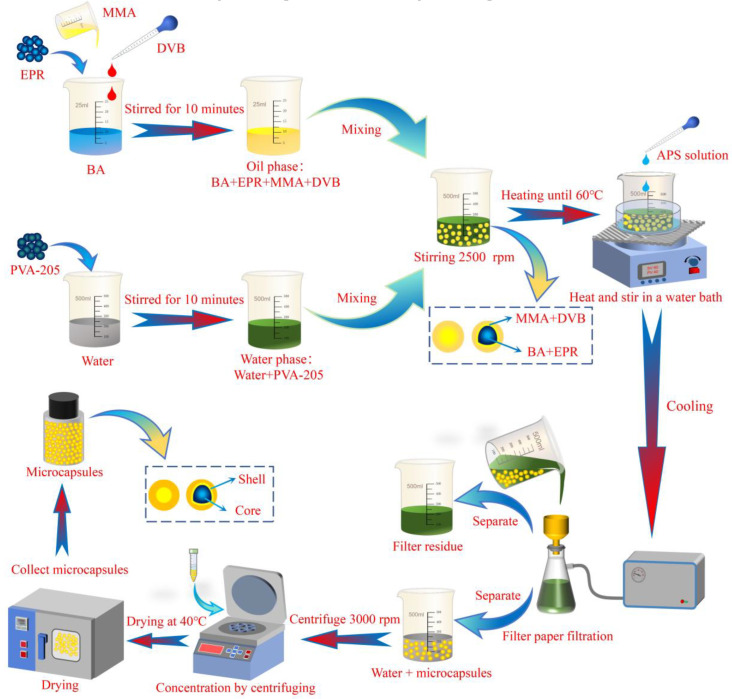
Schematic diagram of microcapsule preparation process.

Then, the suspension is initially filtered through a filter screen to remove larger particles generated by local uneven reactions. The emulsion is then finely filtered using a suction filtration device. The filtered paper is repeatedly rinsed with deionized water to obtain deionized-water-containing microcapsules. The deionized-water-containing microcapsules are then centrifuged using a high-speed centrifuge (HSC, Anting Scientific Instrument Factory, Shanghai, China, Model: KA-1000), and the upper clear liquid is removed with a rubber bulb pipette to obtain deionized water containing a high concentration of microcapsules. Finally, the deionized water containing a high concentration of microcapsules is poured into the evaporation dish, placed in an electric temperature-controlled oven (ETO, Tianyu Plastic Instrument Factory, Shaoxing, China, Model: XMTA-L-6000), and dried at 40 °C for 5 h to obtain the dried microcapsule powder and collect it. The preparation process of the microcapsules is shown in Figure 2.

### 2.4. Experimental Design

In the preparation of microcapsules via interfacial polymerization, factors such as the oil-to-water ratio, emulsifier dosage, emulsification speed, initiator concentration, and core-to-shell ratio play a crucial role. These factors not only determine whether microcapsules can be successfully prepared but also have an important impact on the physical and structural properties of the microcapsules. In order to obtain microcapsules with optimal properties, the effect of each factor on microcapsules was investigated by one-way experiments. Based on the order of study, the optimal parameters for each factor were applied in subsequent experiments, ultimately determining the best process parameters. In the experiment, the amount of aqueous phase solvent was fixed at 200 mL. The remaining experimental parameters are detailed in Table 1. Samples 1, 2, and 3 were used to analyze the effect of the oil-to-water ratio; samples 4, 5, and 6 to analyze the effect of emulsifier dosage; samples 7, 8, and 9 to analyze the effect of emulsification speed; samples 10, 11, and 12 to analyze the effect of initiator dosage; and samples 11, 13, and 14 to analyze the effect of the core-to-shell ratio.

### 2.5. Characterization Methods of Microcapsules

Optical microscopy (OM, Carl Zeiss AG, Oberkochen, Germany, Model: LSM 900) and scanning electron microscopy (SEM, Thermo Fisher Scientific Inc., Waltham, MA, USA, Model: APREO-2S) were used to observe the reaction conditions in the emulsion under different process parameters and preparation stages and to capture surface images of the microcapsules. Nano Measurer 1.2.5 software was employed to collect particle size data for both the microcapsules and small oil droplets. A Fourier transform infrared spectrometer (FT-IR, Thermo Fisher Scientific Inc., Waltham, MA, USA, Model: IS50) was used to analyze the monomers of the shell material, crosslinkers, core material, polymer wall material, and microcapsules. The testing range was 500–4000 cm^−1^, with 32 scans and a resolution of 4 cm^−1^ to obtain the FT-IR spectra of each material. Thermogravimetric analysis (TGA, Nippon Seiko Kabushiki Kaisha, Tokyo, Japan, Model: 6300) was conducted on the core material, polymer wall material, and microcapsules. The analysis was performed in an air atmosphere with a heating rate of 10 °C/min, within a temperature range of 25 °C to 600 °C, to obtain the TGA curves. In a comparative experiment, epoxy coatings containing 8 wt% microcapsules and those without microcapsules were uniformly applied to carbon steel substrates. The coatings were subjected to scratch testing, and the self-healing performance of the microcapsules was verified by comparing the self-repair effects at the scratched areas of both coatings.

## 3. Results and Discussions

### 3.1. Effect of Process Parameters on the Characteristics of Microcapsules

#### 3.1.1. Effect of Oil-to-Water Ratio, Emulsifier Dosage, and Emulsification Speed on the Particle Size of Microcapsules

The particle size of microcapsules is crucial to the performance of the coating. A particle size that is too large may lead to uneven dispersion of the microcapsules in the coating, resulting in a rough coating appearance and reduced adhesion to the substrate. On the other hand, a particle size that is too small may result in insufficient release of the healing agent, compromising the self-healing effect. Therefore, in this study, the optimal particle size for the microcapsules is set at 35 μm, with a permissible range of 25 μm to 45 μm, to ensure excellent dispersion and functionality of the coating. To successfully prepare microcapsules that meet the desired particle size, it is necessary to investigate the various factors that influence microcapsule size.

**Figure 3:**
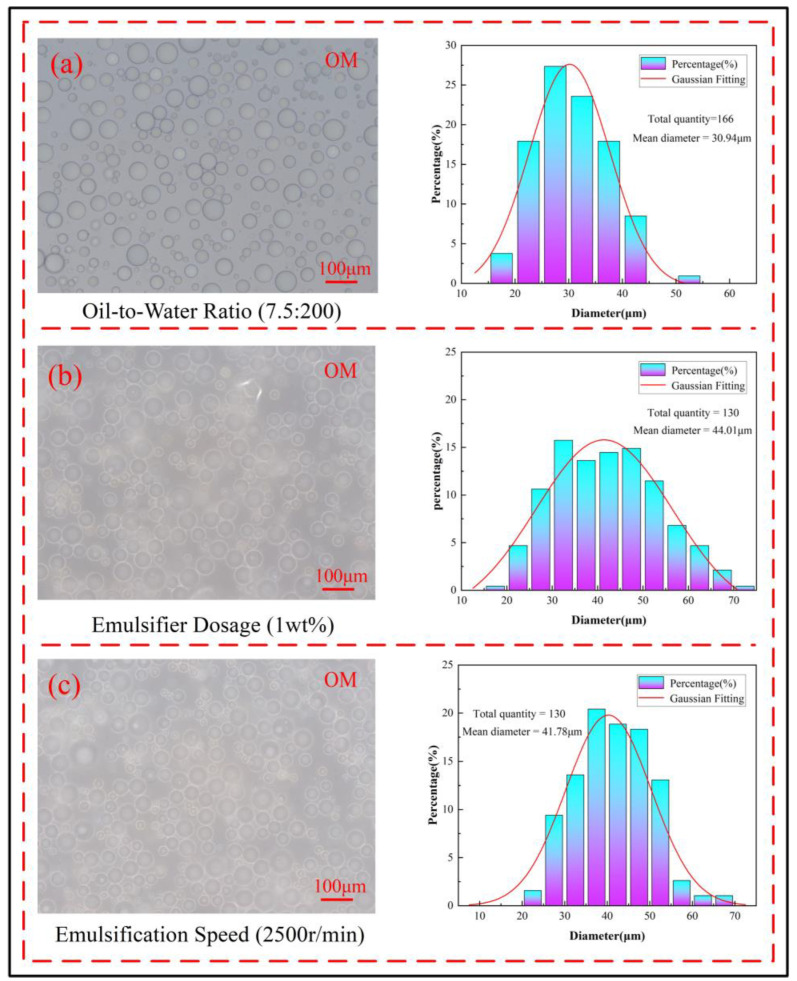
Particle size distribution of microcapsules at the optimal process parameters (**a**) oil-to-water ratio 7.5:200, (**b**) emulsifier dosage 1 wt%, (**c**) emulsification speed 2500 r/min.

In the preparation of microcapsules via interfacial polymerization, factors such as the oil-to-water ratio, emulsifier dosage, and emulsification speed control the particle size of the microcapsules. The oil-to-water ratio determines the dispersion degree of the oil phase in the water phase. If the oil-to-water ratio is too high, the small oil droplets in the emulsion become densely packed, leading to aggregation and rupture. If the oil-to-water ratio is too low, the oil droplets are sparsely distributed, resulting in wasted experimental resources. The particle size distribution of small oil droplets at the optimal oil-to-water ratio is shown in Figure 3a, and the emulsifier dosage determines the stability of the emulsion. An excessive amount of emulsifier can overly stabilize the emulsion, leading to the aggregation or settling of the small oil droplets, while an insufficient amount of emulsifier will result in an unstable emulsion system and difficulty in forming small oil droplets. The particle size distribution of small oil droplets at the optimal emulsifier dosage is shown in Figure 3b; the emulsification speed determines the particle size and uniformity of the small oil droplets. Generally, at low emulsification speeds, the resulting small oil droplets have larger and more uneven particle sizes. As the emulsification speed increases, the cutting frequency within the same time frame increases, causing the particle size of the small oil droplets to gradually decrease and the distribution to become more uniform. However, excessively high emulsification speeds can lead to the formation of excessively small oil droplets. The particle size distribution of the small oil droplets at the optimal emulsification speed is shown in Figure 3c.

#### 3.1.2. Effect of Initiator Dosage on Shell Structure

During the polymerization reaction, the amount of initiator APS determines the polymerization rate of the microcapsule shell, which in turn affects the shell structure of the microcapsules. An appropriate amount of initiator can allow the polymerization reaction to proceed steadily, resulting in higher sphericity microcapsules. When the amount of initiator is too high, the excessively fast polymerization rate may lead to lower sphericity of the microcapsules after the reaction. Conversely, when the initiator concentration is too low, it can result in a polymerization reaction that is too slow or incomplete shell material polymerization, significantly reducing the yield of microcapsules.

**Figure 4:**
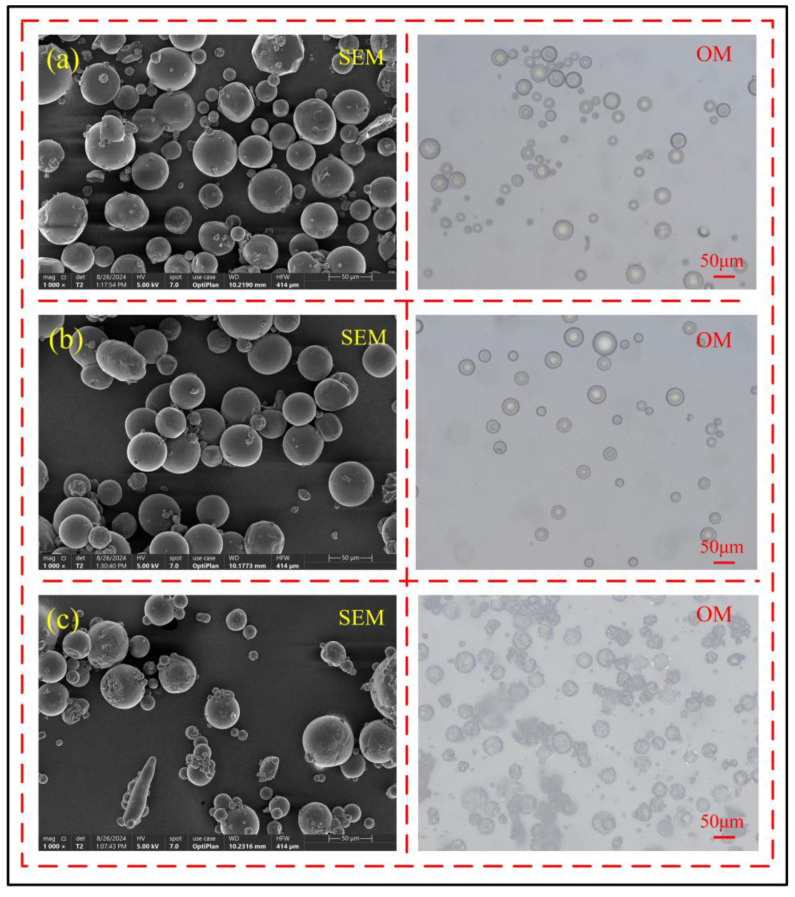
Effect of initiator dosage on the shell structure of microcapsules, (**a**) 2 g, (**b**) 2.5 g, (**c**) 3 g.

When the amount of initiator is 2 g, as shown in Figure 4a, the number of microcapsules in the emulsion is small, the sphericity is average, and the molding rate is low, indicating that the polymerization reaction rate is too slow and the polymerization reaction is incomplete; when the amount of initiator increases to 2.5 g, as shown in Figure 4b, the number of microcapsules in the emulsion significantly increases, and the sphericity is good, indicating that the reaction rate is relatively stable at this time. When the initiator is further increased to 3 g, as shown in Figure 4c, the sphericity of the microcapsules decreases, and the surface is rough, indicating that the amount of initiator is too much, leading to a reaction rate that is too fast. Therefore, the best polymerization reaction effect occurs when the amount of initiator is 2.5 g.

#### 3.1.3. Effect of Core-to-Shell Ratio on Shell Structure and Morphology

The core–shell ratio of microcapsules determines the thickness of the shell, which in turn affects the surface morphology of the microcapsules, such as sphericity and surface smoothness. An appropriate core–shell ratio can ensure that the microcapsule shell forms uniformly, has good sphericity, and has a smooth surface. When the core–shell ratio is too high, the shell material is insufficient, resulting in a thin shell wall that is not stable enough, making it prone to damage or deformation. Some microcapsules may fail to form a complete structure due to insufficient shell material. When the core–shell ratio is too low, there is too much shell material, leading to a thick shell wall that may develop wrinkles due to accumulation, resulting in a rough appearance and affecting its performance.

**Figure 5:**
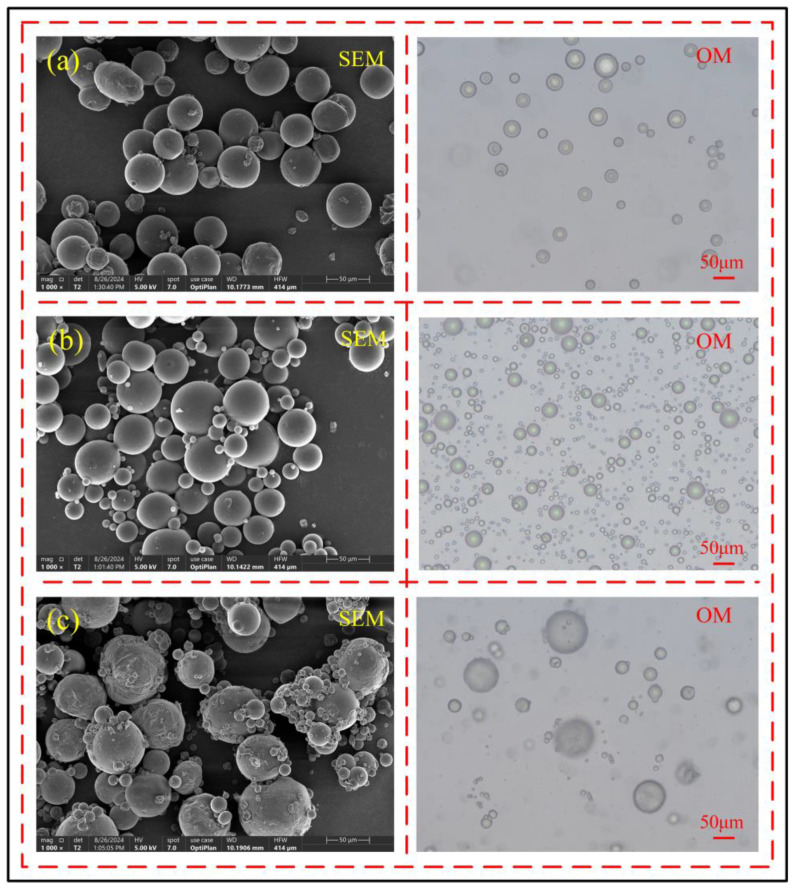
Effect of core-to-shell ratio on the shell structure and morphology of microcapsules, (**a**) 8:1, (**b**) 8:2, (**c**) 8:3.

Through observations using scanning electron microscopy and optical microscopy, it can be found that when the core–shell ratio is 8:1, as shown in Figure 5a, although most microcapsules have formed a complete structure, their sphericity is generally poor, the shell is relatively thin, and its light shielding capability is insufficient. The core material inside the microcapsules can be seen through the light source of the optical microscope, indicating that the core–shell ratio is too large at this time. When the core-to-shell ratio was increased to 8:2, as shown in Figure 5b, all microcapsules had formed complete structures, the shell material thickness increased significantly, and the optical microscope’s light source could no longer penetrate the microcapsules. The microcapsules exhibited high sphericity and smooth surfaces, indicating that the core-to-shell ratio was more appropriate. However, when the core-to-shell ratio was further increased to 8:3, as shown in Figure 5c, the sphericity of the microcapsules decreased, their surfaces became rough, and the shell material was unevenly distributed, causing wrinkles due to excessive shell material accumulation. Therefore, the microcapsules with the best shell morphology were obtained at a core-to-shell ratio of 8:2.

### 3.2. Performance Characterization of Microcapsules

#### 3.2.1. Particle Size and Surface Morphology

The reaction conditions in the emulsion at different process parameters and preparation stages, as well as the prepared microcapsules, were observed and analyzed using optical microscopy and scanning electron microscopy. The optimal process parameters for microcapsule preparation were determined as follows: oil-to-water ratio of 7.5:200, emulsifier concentration of 1 wt%, emulsification speed of 2500 r/min, initiator amount of 2 g, and core-to-wall ratio of 4:1.

**Figure 6:**
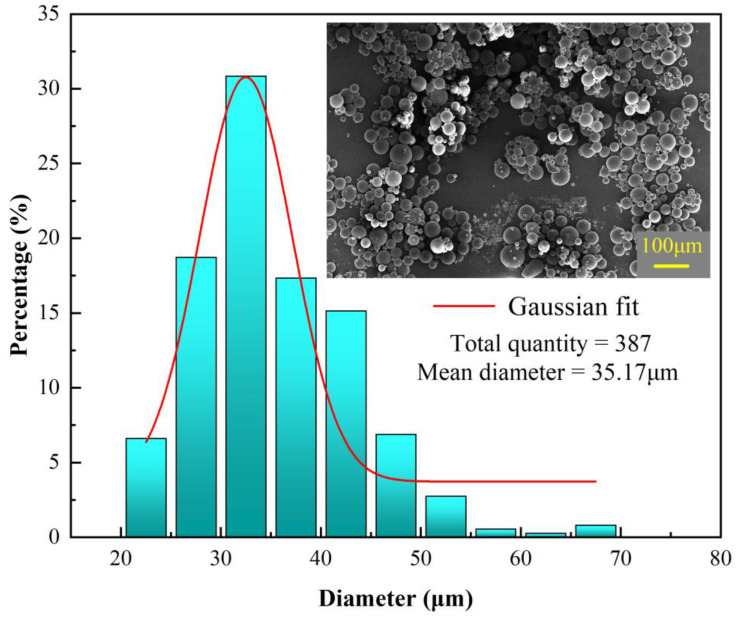
Particle size distribution of microcapsules under optimal preparation process parameters.

As shown in Figure 6, the microcapsules under this parameter present a uniform particle size and have good sphericity, indicating that the reaction process of the microcapsules is relatively stable and the reaction effect is good. The particle size distribution is between 20 μm and 70 μm, with an average diameter of 35.17 μm, showing a good normal distribution. The maximum frequency is around the ideal particle size of 35 μm, accounting for about 30%, while the proportion of microcapsules in the particle size range of 25 μm to 45 μm reaches 80%, indicating that the particle size distribution of the microcapsules is relatively concentrated and well-controlled.

#### 3.2.2. Degree of Crosslinking in Shell Materials

Regarding the crosslinking free radical polymerization reaction between MMA and DVB, when the degree of crosslinking is low, the shell structure of the microcapsules is relatively loose, lacking mechanical strength, and is prone to rupture. As the degree of crosslinking increases, after reaching a moderate degree of crosslinking, the shell exhibits good mechanical strength and toughness, effectively releasing the repair agent; however, when the degree of crosslinking reaches a high level, both the hardness and strength of the shell significantly increase, making it difficult for the core material to be released. Therefore, to ensure the stability of the microcapsule structure and its self-repairing performance, the shell should possess a moderate degree of crosslinking.

**Figure 7:**
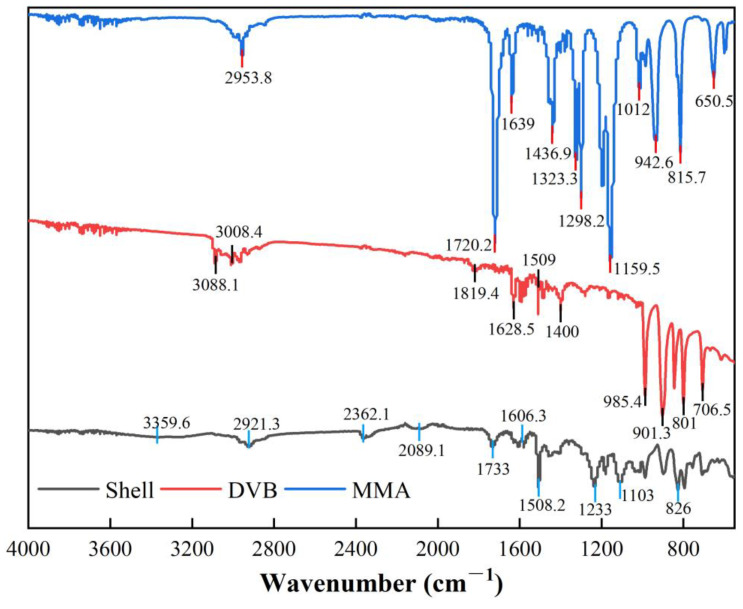
FTIR spectra of MMA, DVB, and crosslinked polymer shell material.

By comparing the FT-IR absorption peaks of MMA, DVB, and the polymer shell material, it can be confirmed that the polymerization reaction has occurred. As shown in Figure 7, the absorption peak of the C=C double bond in the monomers significantly weakens in the polymer, indicating that most of the double bonds have participated in the polymerization and crosslinking reactions. The carbonyl absorption peak of MMA shifts slightly from 1720.2 cm⁻^1^ to 1733 cm⁻^1^, indicating a change in its chemical environment during the reaction, which further supports the formation of the polymer. However, a residual absorption peak at 1606.3 cm⁻^1^ remains in the FT-IR spectrum of the polymer, showing the presence of unreacted double bonds, suggesting that the crosslinking reaction is not fully complete. The characteristic peaks of the carbonyl (C=O) and ester (C-O) groups in MMA remain unchanged during the reaction and can still be observed in the polymer’s spectrum, indicating that the reaction primarily occurred at the vinyl groups, while the ester groups did not participate. Overall, the polymerization reaction has been successfully carried out, with some unreacted double bonds remaining, resulting in a moderate degree of crosslinking that meets the requirements for microcapsule crosslinking.

#### 3.2.3. Efficiency Encapsulation Performance of the Shell

**Figure 8:**
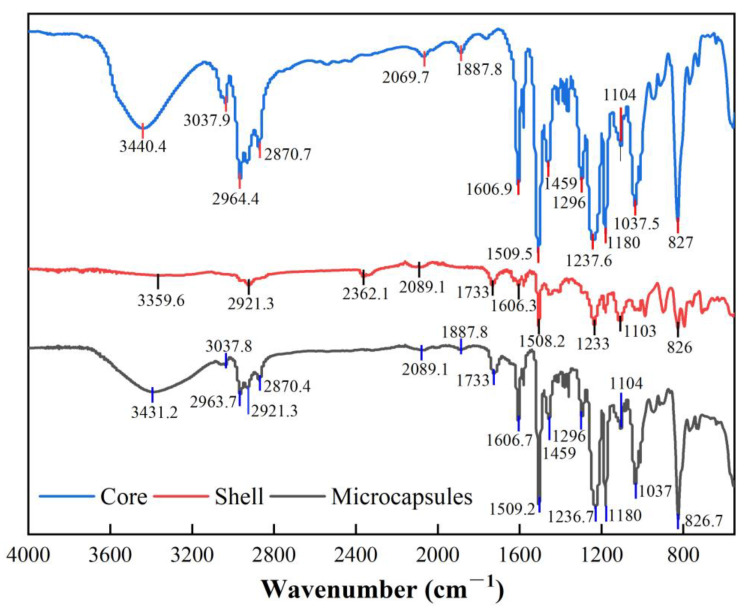
FTIR spectra of core material, shell material, and microcapsules.

By comparing the FT-IR absorption peaks of the core material, shell material, and microcapsules, it can be observed that the shell material encapsulates the core material effectively. As shown in Figure 8, the main characteristic absorption peaks of the shell material appear at 2921.3 cm⁻^1^, 2089.1 cm⁻^1^, and 1733 cm⁻^1^, corresponding to C-H stretching vibrations, C≡N or C=C bond vibrations, and C=O carbonyl stretching vibrations, respectively. In the microcapsule spectrum, these absorption peaks of the shell material are clearly visible, particularly the peak at 1733 cm⁻^1^, which shows high intensity, indicating that the shell material is well-preserved within the microcapsules and has not undergone significant chemical structural changes, demonstrating high stability. At the same time, the characteristic absorption peaks of the core material (e.g., 3440.41 cm⁻^1^ and 3037.9 cm⁻^1^) are also retained in the microcapsules. This suggests that the core material maintains its basic chemical structure under the shell material’s encapsulation and is not completely shielded, indicating the relative independence of the core material within the microcapsules. Furthermore, the coexistence of the characteristic absorption peaks of both the shell and core materials in the microcapsules further confirms their interaction within the microcapsule structure.

#### 3.2.4. Thermal Stability

**Figure 9:**
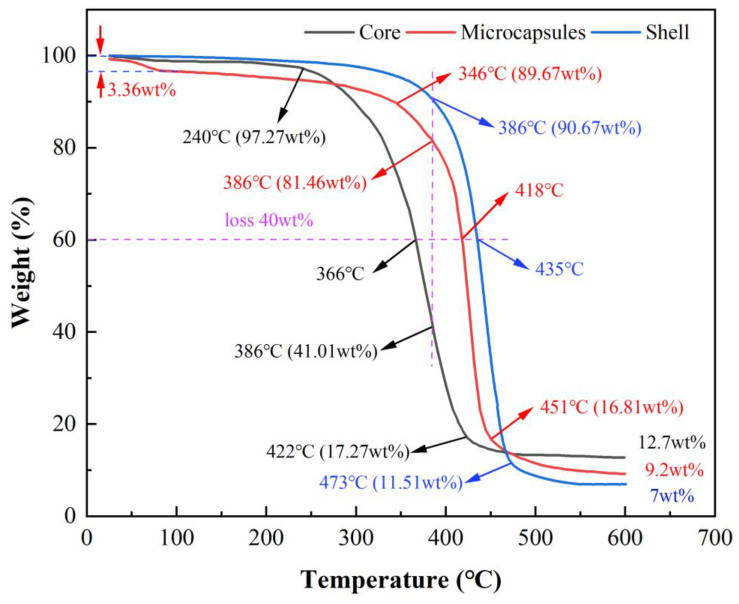
TGA curves of core material, shell material, and microcapsules.

TGA curve analysis shows that the presence of the shell material significantly reduces the decomposition rate of the core material, providing the prepared microcapsules with good thermal stability. As shown in Figure 9, the decomposition rate of the core material increases significantly at 240 °C, with a remaining mass of 97.27 wt%. The decomposition rate slows down at 422 °C, and the remaining mass decreases to 17.27 wt%. In contrast, the shell material’s decomposition rate only starts to increase at 386 °C, with a remaining mass of 90.67 wt%. The rate of loss slows down at 473 °C, and the remaining mass decreases to 11.51 wt%, indicating that the thermal stability of the core material is relatively low, making it prone to decomposition at high temperatures, resulting in significant quality loss, while the thermal stability of the wall material is significantly higher than that of the core material. For the microcapsules, due to the presence of residual moisture that has not been fully dried, the mass decreases by 3.36 wt% during the initial heating stage. At 346 °C, the decomposition rate begins to accelerate, with the remaining mass at 89.67 wt%. At 451 °C, the loss rate slows down, and the remaining mass decreases to 16.81 wt%.

As shown by the dashed line in Figure 9, Comparing the remaining mass at the same temperature, it is observed that when the temperature reaches 386 °C, the mass loss rate of the shell material begins to increase, with a remaining mass of 90.67 wt%. At this temperature, the remaining mass of the core material is only 41.01 wt%, while the microcapsules have 81.46 wt%, indicating that their thermal stability is between that of the core material and the shell material. Further comparison at the same mass loss rate shows that at a 40 wt% loss, the corresponding temperatures are 366 °C for the core material, 435 °C for the shell material, and 418 °C for the microcapsules. At the end of the test, the residue of the core material, shell material, and microcapsules are 12.7 wt%, 7 wt%, and 9.2 wt%, respectively, which further confirms the accuracy of the experimental data.

#### 3.2.5. Self-Healing Performance Verification

Epoxy coatings have excellent adhesion to steel, possess outstanding shielding performance, and can effectively block the penetration of moisture and corrosive substances. In this study, the prepared microcapsules were added to the epoxy coating and evenly applied to the carbon steel substrate, resulting in an epoxy coating with self-healing functionality. To verify the self-healing performance of the microcapsules, the coating was first scratched, and then the epoxy coatings containing 8 wt% microcapsules and those without microcapsules, respectively, were placed in a 3.5 wt% NaCl solution to simulate a marine environment, soaked for 24 h, and the changes in the scratches were observed.

**Figure 10:**
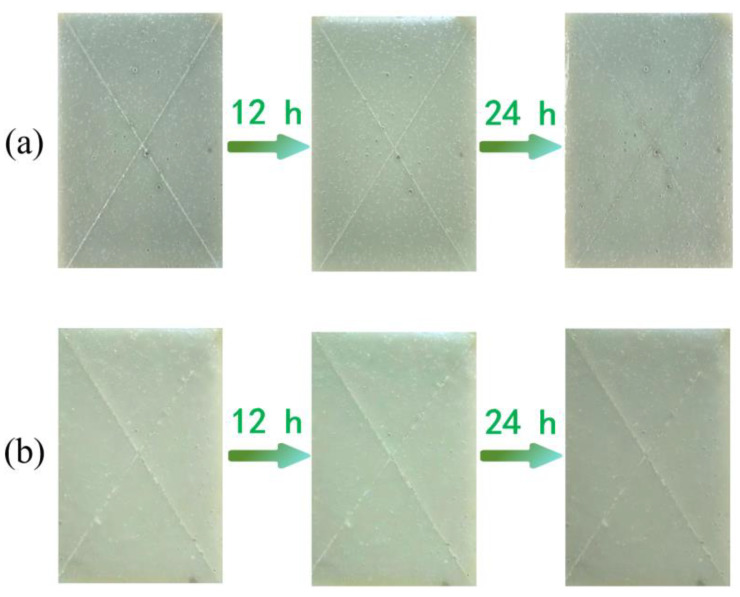
(**a**) Scratch repair effect of self-healing coating containing 8 wt% microcapsules, (**b**) scratch image of coating without microcapsules.

The coating containing 8 wt% microcapsules showed a significant reduction in scratches after being soaked in NaCl solution for 12 h, and after 24 h, the scratches had become very faint, as shown in Figure 10a. In contrast, the coating without microcapsules showed almost no change in scratches after soaking for 12 h and 24 h, as shown in Figure 10b. This indicates that the addition of self-repairing microcapsules significantly enhances the self-repairing performance of the coating.

**Figure 11:**
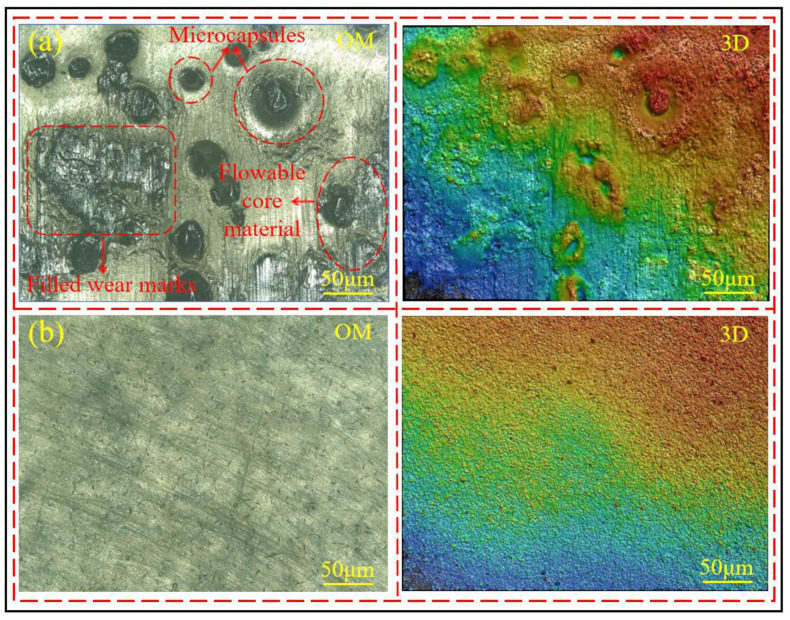
(**a**) Surface morphology of self-healing coating with 8% microcapsules after wear, (**b**) surface morphology of coating without microcapsules after wear.

By comparing the surface morphology of the coating containing 8 wt% microcapsules after friction damage, as shown in Figure 11a, with the surface morphology of the coating without microcapsules after abrasion, as shown in Figure 11b, it can be observed that after the microcapsules are damaged and ruptured, the core material flows out and fills the wear marks. This indicates that the core material inside the microcapsules has good flowability and can promptly repair the damage to the coating.

## 4. Conclusions

In this paper, self-healing microcapsules with EPR as the core material and cross-linked polymer (PMMA-DVB) as the shell were successfully prepared by interfacial polymerization for mitigating the problem of metal corrosion caused by microcracks in steel surface coatings of offshore drilling platforms.

(a) By analyzing the laws of influence of process parameters on the particle size, dispersibility, morphology, and surface characteristics of microcapsules, the optimal process conditions for microcapsule preparation were determined to be an oil-to-water ratio of 7.5:200, an emulsifier content of 1 wt%, an emulsification speed of 2500 r/min, an initiator content of 2.5 g, and a core-to-wall ratio of 4:1.
(b) Under the optimal parameters, FT-IR, TGA, and particle size analysis results show that the prepared microcapsules have a moderate degree of crosslinking in the wall material, which provides good protection to the core material. This significantly improves the thermal stability of the core material and slows down its decomposition process. Compared to the dual-wall self-healing microcapsules prepared in reference [21], the microcapsules prepared in this study have a simpler structure and better thermal stability. The microcapsules exhibit excellent thermal stability up to 346 °C. The particle size of the microcapsules is predominantly concentrated around 35 µm, accounting for approximately 30%, while the particle size range of 25 µm to 45 µm covers 80% of the microcapsules.
(c) The coating scratch experiment shows that the epoxy coating with this microcapsule has good self-healing properties, achieving a good self-healing effect within 24 h. When the microcapsules rupture due to damage to the coating, the repair agent inside can effectively mend the damaged area. This microcapsule has broad application potential in achieving self-healing of surface coatings for marine steel structure engineering equipment.

## Figures and Tables

**Table 1:** Experimental design.

Sample	PVA-205 (g)	MMA (g)	DVB (g)	EPR (g)	BA (g)	APS (g)	Emulsification Speed (r/min)
1	2.55	0.73	0.075	2	4.4	/	2000
2	2.55	1.46	0.15	4	8.8	/	2000
3	2.55	2.19	0.225	6	13.2	/	2000
4	1.65	0.73	0.075	2	4.4	/	2000
5	2.1	0.73	0.075	2	4.4	/	2000
6	2.55	0.73	0.075	2	4.4	/	2000
7	2.1	0.73	0.075	2	4.4	/	2000
8	2.1	0.73	0.075	2	4.4	/	2500
9	2.1	0.73	0.075	2	4.4	/	3000
10	2.1	0.73	0.075	2	4.4	2.0	2500
11	2.1	0.73	0.075	2	4.4	2.5	2500
12	2.1	0.73	0.075	2	4.4	3.0	2500
13	2.1	1.46	0.15	2	4.4	2.5	2500
14	2.1	2.2	0.23	2	4.4	2.5	2500

## Data Availability

The data underlying this study are available in the published article; further inquiries can be directed to the corresponding authors.
